# High-Throughput Method for Wide-Coverage and Quantitative Phenolic Fingerprinting in Plant-Origin Foods and Urine Samples

**DOI:** 10.1021/acs.jafc.2c01453

**Published:** 2022-06-15

**Authors:** Raúl González-Domínguez, Ana Sayago, María Santos-Martín, Ángeles Fernández-Recamales

**Affiliations:** † Agrifood Laboratory, Faculty of Experimental Sciences, University of Huelva, 21007 Huelva, Spain; ‡ International Campus of Excellence CeiA3, University of Huelva, 21007 Huelva, Spain

**Keywords:** phenolic compounds, food analysis, liquid chromatography−diode array detector, metabolomics, urine

## Abstract

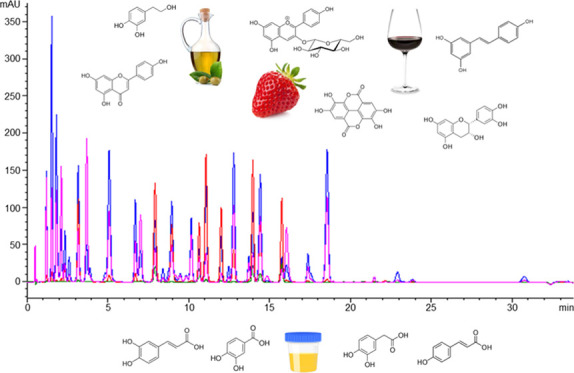

The use of mass spectrometry is currently widespread in polyphenol research because of its sensitivity and selectivity, but its usual high cost, reduced robustness, and nonavailability in many analytical laboratories considerably hinder its routine implementation. Herein, we describe the optimization and validation of a high-throughput, wide-coverage, and robust metabolomics method based on reversed-phase ultra-high-performance liquid chromatography with diode array detection for the identification and quantification of 69 phenolic compounds and related metabolites covering a broad chemical space of the characteristic secondary metabolome of plant foods. The method was satisfactorily validated following the Food and Drug Administration guidelines in terms of linearity (4–5 orders of magnitude), limits of quantification (0.007–3.6 mg L^–1^), matrix effect (60.5–124.4%), accuracy (63.4–126.7%), intraday precision (0.1–9.6%), interday precision (0.6–13.7%), specificity, and carryover. Then, it was successfully applied to characterize the phenolic fingerprints of diverse food products (i.e., olive oil, red wine, strawberry) and biological samples (i.e., urine), enabling not only the detection of many of the target compounds but also the semi-quantification of other phenolic metabolites tentatively identified based on their characteristic absorption spectra. Therefore, this method represents one step further toward time-efficient and low-cost polyphenol fingerprinting, with suitable applicability in the food industry to ensure food quality, safety, authenticity, and traceability.

## 1 Introduction

Phenolic compounds are secondary metabolites exclusively synthesized by plants, so they are ubiquitous in plant-origin foods (e.g., fruits, vegetables, legumes, cereals, nuts) and beverages (e.g., coffee, tea, wine, beer). The chemical structure of plant phenolics is characterized by the presence of one or more hydroxyl substituents attached to at least one aromatic ring.^1^ In general, phenolic compounds that contain more than one aromatic moiety are referred to as polyphenols, although both terms are often used interchangeably. These phytochemicals can in turn be classified into two main categories according to their structure, namely, flavonoids and non-flavonoid compounds.^1^ Flavonoids refer to various polyphenol classes based on a phenyl–benzopyran skeleton, which, depending on the hydroxylation pattern and oxidation state of the central pyran ring, can result in a wide range of flavonoid subfamilies (e.g., anthocyanins, flavonols, flavones, flavanones, flavan-3-ols, isoflavones). Among non-flavonoids, phenolic acids (e.g., hydroxybenzoic acids, hydroxycinnamic acids), tannins, lignans, and stilbenes are also widely distributed within the plant kingdom. Furthermore, phenolic compounds can normally be found in plant foods both in their free form (i.e., aglycones) or in conjugated forms with sugar residues (i.e., glycosides),^1^ which consequently results in a highly complex secondary metabolome with diverse physicochemical properties and concentration ranges.

Polyphenols and related compounds may contribute to the sensory and nutritional characteristics of plant-based foods, including bitterness, astringency, color, flavor, and oxidative stability.^2^ Furthermore, epidemiological and clinical data suggest that the consumption of polyphenol-rich diets is associated with reduced risk of several chronic diseases, such as obesity, diabetes, cancer, and cardiovascular and neurodegenerative diseases, probably as a consequence of the antioxidant, anti-inflammatory, anti-hyperlipidemic, and prebiotic properties of these bioactive phytochemicals.^1^ In this context, it should be noted that multiple factors can affect the polyphenol content of agrifood products, including the cultivar, geographical origin, cultivation conditions, and food processing technologies. Accordingly, the food industry demands accurate and robust analytical methods to guarantee food quality and safety, as well as to monitor food authenticity and traceability.^3,4^

The analysis of polyphenols is usually accomplished by means of liquid chromatography coupled to spectroscopic or mass spectrometry detectors, although other techniques have also been proposed, such as gas chromatography, capillary electrophoresis, and nuclear magnetic resonance.^5^ Nowadays, reversed-phase liquid chromatography coupled to mass spectrometry (RP-LC-MS) has become the gold standard technique for polyphenol research because of its sensitivity, selectivity, high-throughput capacity, wide-coverage, and the potential to perform reliable identifications.^6,7^ The number of applications involving MS has dramatically increased in the last few years, by considering untargeted metabolomics approaches,^8,9^ targeted analysis of specific polyphenol species,^10−12^ as well as large-scale (semi)targeted screening of phenolic compounds and related metabolites in food matrices^13,14^ and in biological samples.^15−17^ However, MS instruments and consumables, as well as MS-grade reagents and solvents, are often costly and not available in many quality control laboratories from the food industry. Furthermore, the application of MS-based analytical techniques normally requires skilled technicians, and their robustness is lower than that provided by other conventional detection techniques, which thereby hinders its implementation in routine analysis and inter-laboratory comparisons.^18^ As an alternative, ultraviolet–visible (UV/Vis) spectroscopy and diode array detection (DAD) have also traditionally been used for the identification and quantification of polyphenols due to their simplicity, low cost, robustness, and usual availability in most analytical laboratories. The acquisition of full UV/Vis spectra enables the creation of spectral libraries, which facilitates the reliable identification of phenolic compounds and the detection of chromatographic coelutions.^19^ Accordingly, LC–UV/Vis and LC–DAD platforms have widely been reported in food science, and, currently, official methods for the determination of phenolic compounds rely on their use.^20,21^ Nevertheless, existing LC–DAD methods typically focus on only a few phenolic compounds (less than 20–30) from specific food groups and normally require a long analysis time.^22−24^ Therefore, we aimed here to develop a novel high-throughput chromatographic method based on robust, simple, and low-cost spectroscopic detection as an alternative to MS, which is a very current topic of great interest for quality control and authentication purposes in the food industry.

In this study, we describe the optimization and validation of a simple, rapid, and wide-coverage metabolomics method based on reversed-phase ultra-high-performance liquid chromatography with diode array detection (RP-UHPLC–DAD) for the quantitation of 69 phenolic-related compounds, including 20 phenolic acids, 5 phenols, 4 benzaldehydes, 4 furan derivatives, 3 phenylethanoids, 1 tannin, 2 stilbenes, and 30 flavonoids. The method was applied to various food (strawberry, red wine, olive oil) and biological (urine) matrices as a case study to evaluate its performance in real samples.

## 2 Materials and Methods

### 2.1 Reagents and Samples

Sodium hydroxide, dimethyl sulfoxide (DMSO), and HPLC-grade acetonitrile, methanol, and formic acid were purchased from Sigma-Aldrich (Steinheim, Germany). Ultrapure water was obtained using a Milli-Q Gradient system (Millipore, Watford, U.K.). Analytical purity standards of benzoic acid, 4-hydroxybenzoic acid, 3,4-dihydroxybenzoic acid, vanillic acid, gallic acid, methylgallate, ethylgallate, syringic acid, phenylacetic acid, 4-hydroxyphenylacetic acid, 3,4-dihydroxyphenylacetic acid, *trans*-cinnamic acid, *o*-coumaric acid, *m*-coumaric acid, *p*-coumaric acid, caffeic acid, ferulic acid, 3-caffeoylquinic acid (chlorogenic acid), sinapic acid, 3-phenylpropionic acid, 3-(4-hydroxyphenyl)propionic acid, benzaldehyde, 4-hydroxybenzaldehyde, 3,4-dihydroxybenzaldehyde, vanillin, syringaldehyde, 4-methylcatechol, 4-ethylphenol, 4-vinylphenol, phenethyl alcohol, eugenol, methoxyeugenol, furfuryl alcohol, furfural, 5-(hydroxymethyl)furfural, 2,5-dimethyl-4-hydroxy-furanone (furaneol), 2,5-dimethyl-4-methoxy-furanone (mesifurane), catechin, epicatechin, epicatechin gallate, epigallocatechin gallate, tyrosol, ellagic acid, naringenin, naringenin 7-O-neohesperidoside (naringin), hesperetin, quercetin, quercetin 3-O-rutinoside (rutin), kaempferol, isorhamnetin, morin, apigenin, 2,6-dimethoxybenzoic acid, and bisphenol A were obtained from Sigma-Aldrich (Steinheim, Germany). 4-O-Methylgallic acid, hydroxytyrosol, oleuropein, quercetin 3-O-glucoside (isoquercitrin), quercetin 3-O-galactoside (hyperoside), kaempferol 3-O-glucoside, isorhamnetin 3-O-glucoside, *trans*-resveratrol, *trans*-resveratrol 3-O-glucoside (*trans*-piceid), cyanidin, pelargonidin, peonidin, malvidin, delphinidin 3-O-glucoside (myrtillin), cyanidin 3-O-glucoside (chrysanthemin), pelargonidin 3-O-glucoside (callistephin), petunidin 3-O-glucoside, peonidin 3-O-glucoside, and malvidin 3-O-glucoside (oenin) were from Extrasynthese (Genay, France). Hesperetin 7-O-rutinoside (hesperidin) and luteolin were purchased from Alfa Aesar (Ward Hill, MA), whereas quercetin 3-O-rhamnoside (quercitrin) was from Phytolab (Vestenbergsgreuth, Germany). Individual stock solutions were prepared at 10 000 mg L^–1^ for all of the phenolic compounds and internal standards (2,6-dimethoxybenzoic acid, bisphenol A), except for ellagic acid (5000 mg L^–1^) and anthocyanins (1000 mg L^–1^), using methanol (for phenolic acids, simple phenols, benzaldehydes, furan derivatives, phenylethanoids, flavan-3-ols, anthocyanins, and internal standards), methanol/DMSO 75:25 (for other flavonoids and stilbenes), or 1 M sodium hydroxide (for ellagic acid) as the solvent (Table S1). From these stock solutions, three multimetabolite working solutions were prepared at 100 mg L^–1^ in water/acetonitrile (1:1, v-v) containing phenolic acids, simple phenols, benzaldehydes, furan derivatives, phenylethanoids, flavan-3-ols, and ellagic acid (solution A); flavonoids (except flavan-3-ols and anthocyanins), stilbenes, methylgallate, ethylgallate and 4-methylcatechol (solution B); and anthocyanins (solution C). These multimetabolite working solutions were used to build the calibration curves by serial dilution in ultrapure water and to spike samples for validation purposes. All of the stock and working solutions were stored at −20 °C until use.

Strawberry, red wine, and extra virgin olive oil samples were purchased from a local market. First morning void human urine samples were collected from healthy volunteers following the principles contained in the Declaration of Helsinki. All samples were stored at −20 °C until use.

### 2.2 Sample Extraction

The food and biological samples under study were extracted following previously optimized methods, with minor modifications.^11,25^ Briefly, 1 mL of methanol/water (80:20, v-v) was added to 0.5 g of olive oil in an Eppendorf tube and vigorously vortexed for 1 min.^25^ The mixture was then centrifuged at 10 000*g* for 10 min, and the supernatant was transferred to a new tube. Finally, the extract was washed twice by adding 0.5 mL of hexane, vortexing for 1 min, and centrifuging at 10 000*g* for 10 min. For strawberry, samples were first homogenized using a kitchen mixer, and a 0.2 g aliquot of the homogenate was then mixed with 1 mL of 1% formic acid in methanol (v:v).^11^ After sonication for 15 min using an ultrasonic bath, the sample was centrifuged at 10 000*g* for 10 min, and the supernatant was transferred to a new tube. Red wine and urine samples were directly injected into the LC system without any prior extraction. Internal standards (2,6-dimethoxybenzoic acid, bisphenol A) were added to the sample extracts to reach a final concentration of 20 mg L^–1^. All samples were filtered through 0.22 μm PTFE filters before analysis.

### 2.3 Chromatographic Analysis of Phenolic Compounds

Analyses were carried out in an Agilent 1260 ultra-high-performance liquid chromatography system equipped with a binary pump, autosampler, and diode array detector (Agilent Technologies, Santa Clara, CA). The chromatographic separations were performed by injecting 5 μL of the sample into a Kinetex EVO C18 column (100 mm × 2.1 mm, 2.6 μm) thermostated at 40 °C and equipped with a SecurityGuard ULTRA Cartridge UHPLC C18 from Phenomenex (Torrance, CA). Two mobile phase sets were employed for the analysis of anthocyanin and non-anthocyanin compounds, which were delivered at a 0.5 mL min^–1^ flow rate. The separation of anthocyanins was achieved using 5% formic acid in water (A) and 5% formic acid in acetonitrile (B) as the mobile phases and applying the following gradient program: 0–10 min, 0–15% B; 10–14 min, 15–100% B; 14–18 min, 100% B; and 18–23 min, 0% B. For analyzing other phenolic compounds, mobile phases consisted of 0.1% formic acid in water (A) and acetonitrile (B), which were delivered as follows: 0–3 min, 0% B; 3–16 min, 0–12% B; 16–16.5 min, 12–16% B; 16.5–21 min, 16% B, 21–25 min, 16–20% B; 25–30 min, 20% B; 30–31 min, 20–100% B; 31–34 min, 100% B; and 34–39 min, 0% B. For quantitative purposes, the detection was carried out by monitoring five different wavelengths (Table S2): 280 nm for most phenolic acids, phenols, benzaldehydes, furan derivatives, phenylethanoids, flavan-3-ols, flavanones, and internal standards; 260 nm for ellagic acid and a few simple phenolic compounds (i.e., 4-hydroxybenzoic acid, 3,4-dihydroxybenzoic acid, vanillic acid, 4-O-methylgallic acid, phenylacetic acid, phenylpropionic acid, phenethyl alcohol, 4-vinylphenol, benzaldehyde); 320 nm for hydroxycinnamic acids (except *trans*-cinnamic acid, *o*-coumaric acid, and *m*-coumaric acid) and stilbenes; 360 nm for flavonols, and flavones; and 520 nm for anthocyanins. Complementarily, full UV/Vis spectra were acquired within the wavelength range of 190–600 nm. To identify phenolic compounds, a spectral library containing retention times and UV/Vis spectra was created by analyzing available commercial standards.

### 2.4 Analytical Validation

The RP-UHPLC–DAD method was validated in terms of linearity, sensitivity, matrix effect, accuracy, intra- and interday precision, specificity, and carryover, according to the guidelines established by the US Food and Drug Administration (FDA).^26^ The linearity was evaluated by analyzing 12-point calibration curves within the concentration range of 0.01–100 mg L^–1^, which were prepared both in solvent and in food matrix (i.e., red wine, olive oil, strawberry homogenate). All of the points of the calibration curves contained 20 mg L^–1^ of 2,6-dimethoxybenzoic acid and bisphenol A as the internal standards. The limits of quantification (LOQ) were estimated from calibration curves using the formula 10 × *Sy*/*S*, where *Sy* refers to the standard deviation of *y*-intercepts and *S* to the slope of the curve.^27^ To assess the matrix effect (ME), the slopes of the calibration curves prepared in solvent and in pre-extracted food samples were compared using the formula [100 × slope_food_/slope_solvent_]. The instrumental accuracy was determined by spiking ultrapure water and pre-extracted food samples with all of the phenolic compounds under study at three concentration levels (0.5, 5, 50 mg L^–1^), which were in turn analyzed in triplicate. The accuracy was computed considering the concentration detected in blank samples using the formula [100 × (concentration_spiked sample_ – concentration_blank sample_)/spiked concentration]. Intra- and interday precisions were assessed by computing the relative standard deviations obtained from analyzing samples spiked at three concentration levels (0.5, 5, 50 mg L^–1^) five times within the same day as well as on three consecutive days, respectively. To evaluate the specificity, we tested the absence of interferences in extraction blanks (i.e., extracts prepared by replacing the food sample with water during the extraction process), computed the retention time variability in solvent and in spiked food samples along a 3-day analytical run, and compared the UV/Vis spectra acquired in spiked samples with those obtained for pure standard solutions. The carryover was checked by analyzing blank water after injecting samples spiked at 50 mg L^–1^ for all of the phenolic compounds under study.

## 3 Results and Discussion

### 3.1 Optimization of the RP-UHPLC–DAD Method

The aim of this work was to develop a rapid, simple, and comprehensive RP-UHPLC–DAD method suitable for the analysis of a broad spectrum of polyphenols and related metabolites. To maximize the coverage and applicability of the method, we considered not only multiple phenolic compounds that are expected to be ubiquitous to most plant-origin foods (e.g., benzoic acids, cinnamic acids, flavonols) but also other characteristic polyphenol classes from specific food groups (e.g., stilbenes, phenylethanoids), with special focus on the main crops grown in Spain and, particularly, in the province of Huelva (i.e., berry and citrus fruits, olive oil, wine). Thus, a total of 74 phenolic compounds were initially included in the optimization and validation of the method, encompassing 22 phenolic acids (9 benzoic acids, 3 phenylacetic acids, 8 cinnamic acids, and 2 phenylpropionic acids), 5 benzaldehydes, 6 phenolic alcohols, 5 furan derivatives, 3 phenylethanoids, 1 tannin, 2 stilbenes, and 30 flavonoids (4 flavan-3-ols, 4 flavanones, 10 flavonols, 2 flavones, and 10 anthocyanins).

Considering the large physicochemical diversity of the target compounds, careful optimization of the chromatographic conditions was critical to get optimal analytical performance as a compromise between peak resolution, sensitivity, and total run time (i.e., high-throughput capacity). First, we tried to develop a single chromatographic method able to simultaneously resolve the entire set of phenolics under analysis. Low pH mobile phases were required to maintain anthocyanins in their flavylium cationic form, which is necessary to improve their chromatographic resolution and to maximize their UV/Vis absorption at 520 nm.^28^ However, these highly acidic concentrations impeded the adequate separation of other polyphenols, so we finally decided to optimize two different chromatographic methods for analyzing anthocyanin and non-anthocyanin compounds in separate runs, as commonly reported in the literature.^28^ After a preliminary screening of various reversed-phase stationary phases, we decided to use a Kinetex EVO C18 column because of its stability at very low pH and excellent resolving power. Using this column, the best separation and peak shapes for anthocyanins was achieved by adding 5% formic acid to both mobile phases and by applying a rapid two-gradient program (0–10 min, 0–15% B; 10–14 min, 15–100% B). In contrast, mobile phases consisting of 0.1% formic acid in water (A) and acetonitrile (B) provided the best chromatographic performance in terms of peak area, peak symmetry, and resolution for the rest of phytochemicals. To properly separate the wide range of non-anthocyanin compounds considered in this work, a multigradient elution program was optimized as follows. The column was first maintained at a high aqueous proportion (100% A) for 3 min to resolve highly polar phenolic compounds (e.g., gallic acid, 3,4-dihydroxybenzoic acid, furan metabolites). A slow gradient within 13 min, ranging from 0 to 12% organic mobile phase, was then applied to separate most phenolic acids, benzaldehydes, and flavan-3-ols. Afterward, flavonoid glycosides and stilbenes were eluted in isocratic mode (16.5–21 min, 16% B) to achieve good resolution between the various chemically analogous species under study. Finally, the content of the organic mobile phase was raised to 20% for eluting more retained metabolites, mainly flavonoid aglycones. To ensure reliable reproducibility, the equilibration time between injections was set at 5 min for both chromatographic methods, which considerably minimized the inter-sample variability in retention times and peak areas and thereby facilitated the unequivocal identification of the peaks of interest in complex samples and improved the analytical accuracy. Moreover, different flow rates (0.4–0.6 mL min^–1^), column temperatures (25–40 °C), and injection volumes (2–10 μL) were also tested to maximize chromatographic resolution and sensitivity, which were finally set at 0.5 mL min^–1^, 40 °C and 5 μL, respectively. Under these conditions, coelutions were observed between various flavonol glycosides (i.e., quercetin 3-O-rutinoside and quercetin 3-O-glucoside, quercetin 3-O-rhamnoside, and kaempferol 3-O-glucoside), and although several attempts were made to favor their separation (e.g., use of methanol as the organic mobile phase, addition of ammonium salts as a modifier), none of the modifications that were tested yielded better results. To avoid compromising the high-throughput capacity of the method, we decided not to increase chromatographic run times and thus quantify these closely coeluting compounds as a sum of both species. For other compounds with similar RTs (e.g., cyanidin and malvidin 3-O-glucoside), partial coelutions could be solved by sample dilution, thus allowing their separate quantification.

With regards to the spectroscopic method, absorption spectra acquired at 280 nm enabled the detection of most phenolic compounds under study, whereas other wavelengths provided increased sensitivity and/or selectivity for the analysis of specific phenolic classes: 260 nm for ellagic acid, 4-hydroxybenzoic acid, 3,4-dihydroxybenzoic acid, vanillic acid, 4-O-methylgallic acid, and 4-vinylphenol; 320 nm for hydroxycinnamic acids (i.e., *p*-coumaric acid, caffeic acid, ferulic acid, 3-caffeoylquinic acid, and sinapic acid) and stilbenes; 360 nm for flavonols, and flavones; and 520 nm for anthocyanins. However, none of these wavelengths yielded enough sensitivity for the detection of several nonhydroxylated phenolic compounds (i.e., phenylacetic acid, phenylpropionic acid, phenethyl alcohol, benzaldehyde, furfuryl alcohol), so they were removed from the final set of target metabolites.

**Figure 1:**
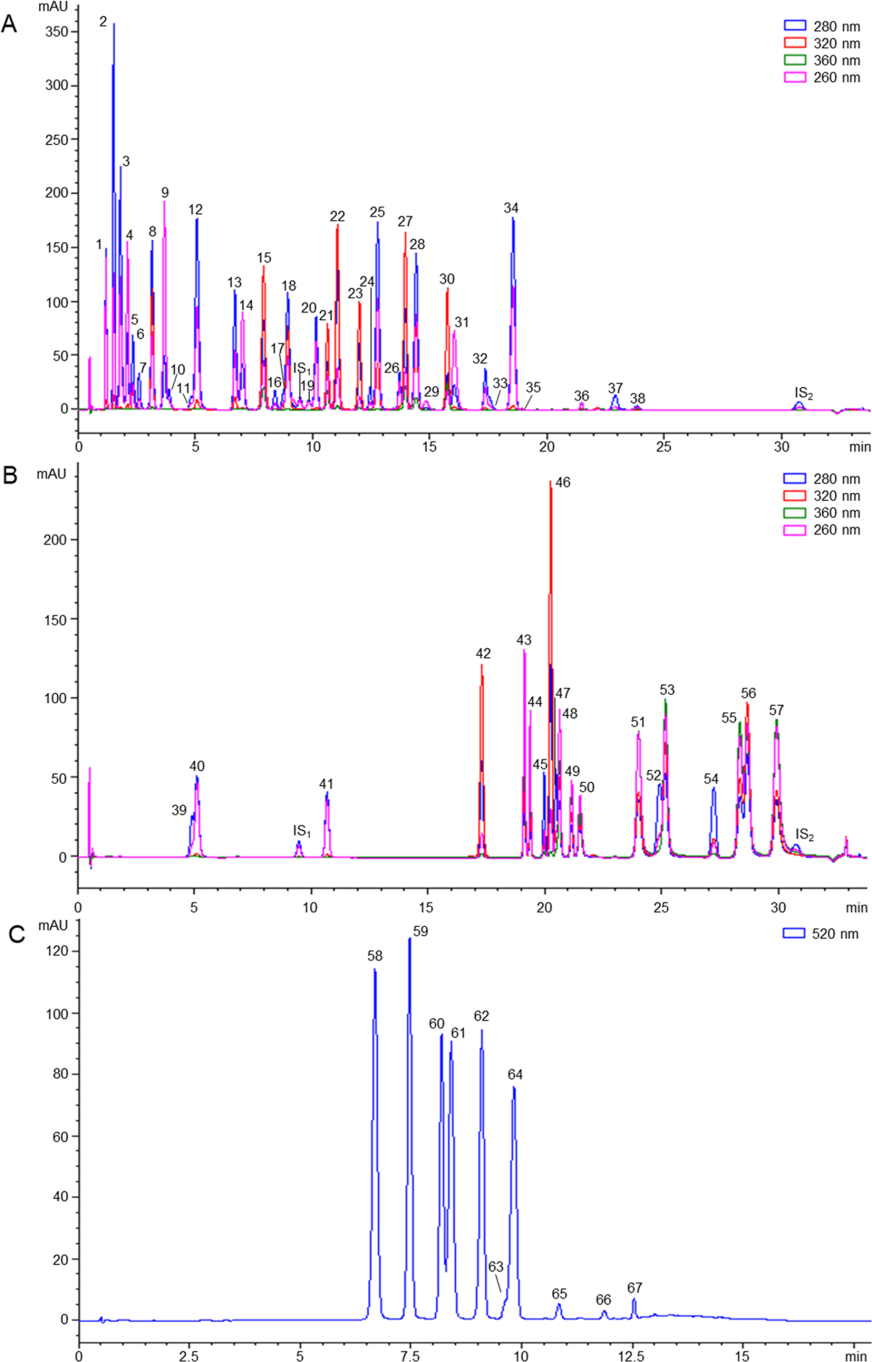
Representative RP-UHPLC–DAD chromatograms obtained by analyzing the three multimetabolite standard mixtures (i.e., solutions A, B, and C, at 25 mg L^–1^). 1: gallic acid; 2: 5-(hydroxymethyl)furfural; 3: furfural; 4: 3,4-dihydroxybenzoic acid; 5: hydroxytyrosol; 6: 2,5-dimethyl-4-hydroxy-furanone; 7: 3,4-dihydroxyphenylacetic acid; 8: 3,4-dihydroxybenzaldehyde; 9: 4-hydroxybenzoic acid; 10: tyrosol; 11: 4-hydroxyphenylacetic acid; 12: 4-hydroxybenzaldehyde; 13: 2,5-dimethyl-4-methoxy-furanone; 14: vanillic acid; 15: caffeic acid; 16: catechin; 17: 3-(4-hydroxyphenyl)propionic acid; 18: vanillin; 19: benzoic acid; 20: syringic acid; 21: 3-caffeoylquinic acid; 22: *p*-coumaric acid; 23: syringaldehyde; 24: epicatechin; 25: *m*-coumaric acid; 26: epigallocatechin gallate; 27: ferulic acid; 28: *o*-coumaric acid; 29: 4-vinylphenol; 30: sinapic acid; 31: 4-O-methylgallic acid; 32: epicatechin gallate; 33: 4-ethylphenol; 34: *trans*-cinnamic acid; 35: ellagic acid; 36: oleuropein; 37: eugenol; 38: methoxyeugenol; 39: 4-methylcatechol; 40: methylgallate; 41: ethylgallate; 42: *trans*-resveratrol 3-O-glucoside; 43: quercetin 3-O-galactoside; 44: quercetin 3-O-rutinoside/quercetin 3-O-glucoside; 45: naringenin 7-O-neohesperidoside; 46: *trans*-resveratrol; 47: hesperetin 7-O-rutinoside; 48: quercetin 3-O-rhamnoside/kaempferol 3-O-glucoside; 49: isorhamnetin 3-O-glucoside; 50: morin; 51: quercetin; 52: naringenin; 53: luteolin; 54: hesperetin; 55: kaempferol; 56: apigenin; 57: isorhamnetin; 58: delphinidin 3-O-glucoside; 59: cyanidin 3-O-glucoside; 60: pelargonidin 3-O-glucoside; 61: petunidin 3-O-glucoside; 62: peonidin 3-O-glucoside; 63: cyanidin; 64: malvidin 3-O-glucoside; 65: pelargonidin; 66: peonidin; and 67: malvidin; IS_1_: 2,6-dimethoxybenzoic acid; IS_2_: bisphenol A.

In summary, the method optimized enables the identification and quantitation of 10 anthocyanins and 59 non-anthocyanin phenolic compounds in total run times of 23 and 39 min, respectively, including washing and equilibration steps (Figure 1). The coverage and high-throughput capacity of this method clearly surpass those shown by previously published LC–DAD-based approaches, which often need longer analysis times (usually ranging from 30 to 120 min) for the determination of fewer polyphenol species (less than 20–30 metabolites).^22−24^ Furthermore, it should be noted that the chromatographic conditions and mobile phases employed here are compatible with MS detection, which would facilitate the migration of the method to LC-MS systems if needed.

### 3.2 Analytical Validation

To guarantee adequate performance for the analysis of real samples, the RP-UHPLC–DAD method was validated using various food matrices of different nature (i.e., aqueous vs fatty samples; solid vs liquid samples) as case study samples, namely strawberry, red wine, and olive oil. Of note, the chromatographic method optimized for anthocyanins was only validated in strawberry and red wine, since these compounds are not expected to be present in olive oil. Method validation parameters, retention times (RT), and the maximum of absorbance for the target phenolic compounds are summarized in Table S2.

The calibration curves, prepared both in solvent and in food matrices, showed linear responses over 4–5 orders of magnitude for most of the phenolic compounds within the concentration range 0.05–100 mg L^–1^ (*R*^2^ > 0.99). However, the linearity was slightly shortened for some metabolites displaying higher limits of quantification. In this respect, it should be noted that most target analytes were quantifiable at sub-ppm levels in all of the matrices under study, with LOQs in the range 0.01–0.1 mg L^–1^. Only a few phenolic alcohols, flavan-3-ols, flavonoid aglycones, and some other compounds (e.g., ellagic acid, oleuropein) presented higher LOQs ranging from 0.1 to 1 mg L^–1^, whereas the lowest sensitivity was obtained for anthocyanin aglycones (LOQs: 1.2–3.6 mg L^–1^). In any case, these LOQs proved to be satisfactory for quantifying the target compounds at the concentration levels that are usually detected in food samples (see Section 3.3). The matrix effect was found to be negligible for most phenolics in the three food products considered here. In general, significant signal suppression (ME: 60–70%) was only observed for a few highly polar metabolites eluting close to the void chromatographic volume (e.g., gallic acid, 5-(hydroxymethyl)furfural). However, some specific compounds were particularly affected by higher matrix effects in at least one of the three food matrices, probably because of their presence at high concentrations in the original sample (e.g., malvidin and malvidin 3-O-glucoside in red wine; 3,4-dihydroxyphenylacetic acid, flavan-3-ols, and pelargonidin in strawberry). Thus, these results evidence that calibration curves prepared in solvent could provide similar performance to that obtained with matrix-matched calibrations, which considerably simplifies the analytical process. Taking this into consideration, the instrumental accuracy was estimated by analyzing ultrapure water and pre-extracted food samples spiked at three concentration levels (0.5, 5, 50 mg L^–1^), and using the calibration curves prepared in solvent for quantification purposes. The accuracy percentages were in general in the range 77.1–126.7%, except for those metabolites suffering from sharpened matrix effects (63.4–75.6%), thereby fulfilling the FDA acceptance criteria. To evaluate the instrumental precision, samples spiked at three concentration levels (0.5, 5, 50 mg L^–1^) were analyzed five times within the same day as well as on 3 consecutive days. The relative standard deviations for intra- and interday precision resulted to be in the ranges 0.1–9.6 and 0.6–13.7%, respectively. Only a few volatile (e.g., furfural) and light/temperature-sensitive (e.g., anthocyanin aglycones) compounds displayed slightly lower interday precisions, but none surpassed the 15% limit established by the FDA. The method specificity was determined by assessing the RT reproducibility along the 3-day analysis run, as well as by comparing the RTs detected in solvent and in the food matrix for each analyte. Interestingly, the RT deviations were below ±3 s for all phenolic compounds, thus demonstrating the stability and robustness of the RP-UHPLC–DAD platform and, consequently, its specificity to differentiate potential interferences. Furthermore, similarity comparisons of the UV/Vis spectra acquired in spiked samples with respect to those obtained for pure standard solutions enabled us to discard the occurrence of coelutions. The injection of extraction blanks at the beginning of the sequence also evidenced the absence of interfering peaks in the chromatographic profile coming from chemicals, labware, and the LC instrument, whereas the analysis of blank water after injecting samples spiked at 50 mg L^–1^ proved that carryover is negligible for all of the target compounds.

Compared with the results previously reported by other authors using similar LC–DAD-based platforms, the method optimized and validated in the present work provides similar, or even enhanced, analytical performance in terms of linearity, sensitivity, accuracy, and precision.^22−24^ Interestingly, the instrumental performance was also comparable to that provided by LC-MS for the analysis of phenolic compounds in food matrices.^10−12^ As expected, only the sensitivity was significantly worsened when using spectroscopic detection, with LOQs being 5–50-fold higher compared to those normally obtained with MS. However, the most remarkable improvement of the method developed here is its high-throughput capacity and wide-coverage, thus enabling the time-efficient and low-cost quantification of a broad range of polyphenols and related phytochemicals in different food products.

### 3.3 Method Application to Real Samples

**Figure 2:**
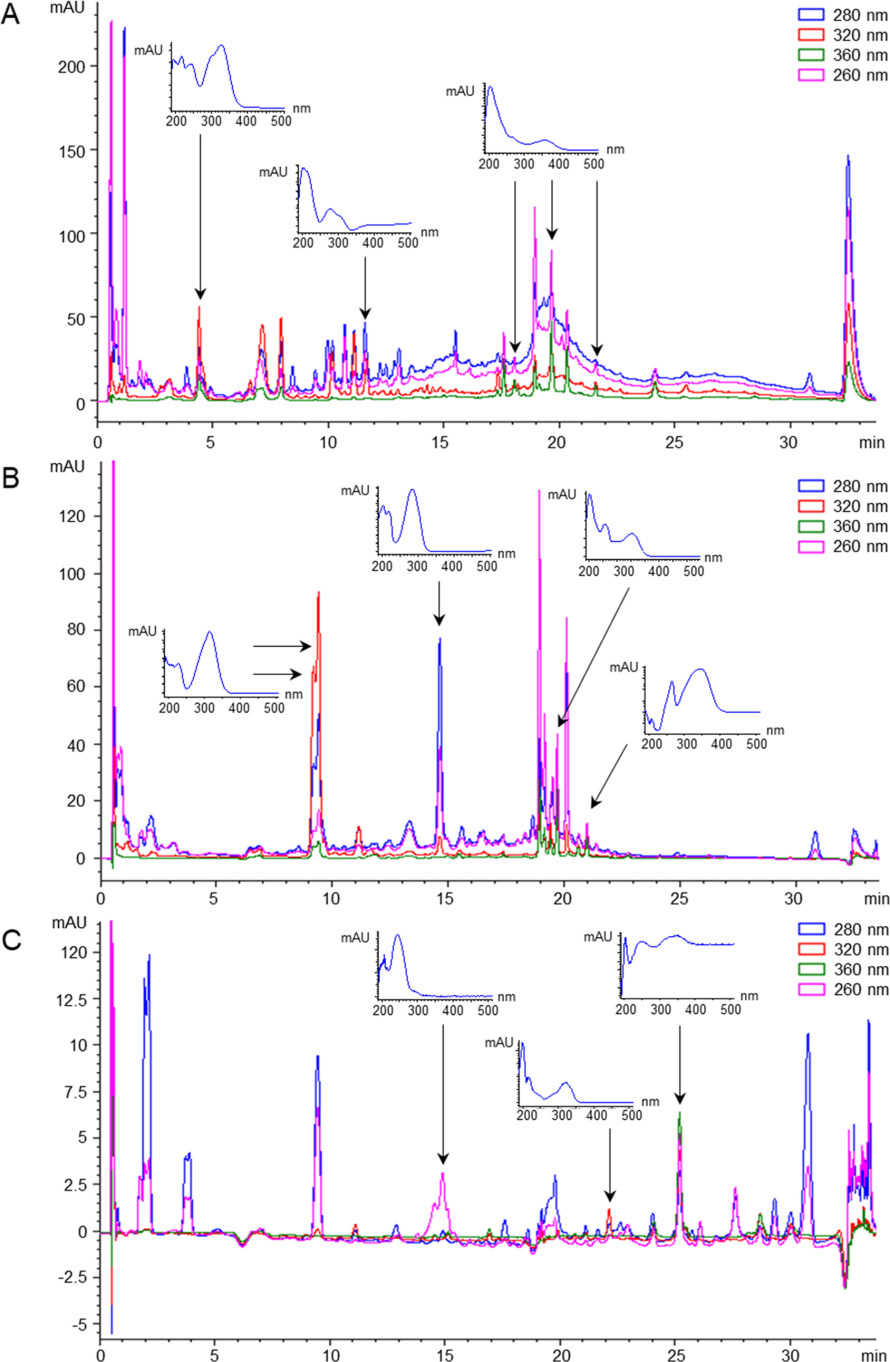
Representative RP-UHPLC–DAD chromatograms obtained by analyzing red wine (A), strawberry (B), and olive oil (C) samples, with UV/Vis spectra for the major unknown peaks that were detected.

The RP-UHPLC–DAD method was successfully applied to investigate the characteristic phenolic fingerprints of olive oil, red wine, and strawberries as case study samples (Table S3). Some metabolites were detected at variable concentrations in all of the food matrices due to their ubiquitous presence in most plant species (e.g., 3,4-dihydroxybenzoic acid, *p*-coumaric acid, catechin, quercetin). Conversely, other phenolic compounds resulted to be food-specific, so they could serve as reliable markers for authenticity purposes and for adulteration detection. Olive oil samples showed high contents of phenylethanoid derivatives (i.e., tyrosol, hydroxytyrosol, oleuropein) and flavones (i.e., luteolin, apigenin).^25^ The phenolic profile of red wine was characterized by several alcohol-related metabolites (i.e, ethylgallate, 4-ethylpehnol), as well as by other grape-origin polyphenols (e.g., malvidin, petunidin, stilbenes).^29^ For strawberries, the most characteristic phytochemicals were pelargonidin derivatives and 2,5-dimethyl-4-hydroxy-furanone, metabolites that are responsible for the distinctive color and aroma of this berry fruit, respectively.^11,30^ Besides these target compounds, which were unambiguously identified and quantified, thanks to the availability of the corresponding standard, we also detected other peaks that could tentatively be identified as various phenolic derivatives based on their characteristic UV/Vis spectra (Figure 2). Phenolic compounds usually show intense UV absorption at 280 and/or 254 nm, which can be accompanied by other distinctive spectral features depending on the chemical class.^31^ For instance, hydroxycinnamic acids are characterized by an additional absorption band at 320 nm, whereas flavonols and flavones absorb at 360 nm. The maximum spectral absorption of stilbenes is located around 300–320 nm. Additionally, colored polyphenols can also absorb in the visible region, such as anthocyanins that show an absorption band at 520 nm, characteristic of reddish substances. These “unknown” compounds identified based on their characteristic spectral features can in turn be semi-quantified using the calibration curves of chemically analogous metabolites for which the standard was available, as previously described.^15,16^ Therefore, this would enable considerably enlarging the coverage of our method beyond the 69 compounds that were initially considered for optimization and validation.

After food ingestion, dietary phytochemicals are metabolized (e.g., phase I/II reactions, gut microbiota biotransformations) and then rapidly excreted, mostly in urine.^32,33^ Therefore, phenolic compounds and related metabolites can serve as suitable biomarkers of food intake.^34^ As a pilot study, we evaluated the potential of the RP-UHPLC–DAD method optimized here to detect these secondary plant metabolites in first morning void human urine samples that were collected from healthy volunteers. Interestingly, the method proved satisfactory performance for the detection and quantification of multiple phenolic acids (e.g., 4-hydroxybenzoic, 3,4-dihydroxybenzoic, gallic, 3,4-dihydroxyphenylacetic, *o*/*m*/*p*-coumaric, and caffeic acids), which have been described as general markers of plant-based food consumption.^35^ In contrast, no flavonoid species were detected in urine, neither in their aglycone form nor as glycoside conjugates. This was totally expected considering the usual low bioavailability of polyphenols, which normally undergo extensive metabolization to yield a myriad of glucuronidated and sulfated metabolites.^32^ Although not tested in the current study, the treatment of urine samples with hydrolytic enzymes would enable the estimation of metabolized flavonoids as aglycone equivalents,^36^ thus maximizing the applicability of our RP-UHPLC–DAD method in nutrimetabolomics research.

In conclusion, the novel method optimized and validated here enables for the first time the comprehensive and quantitative fingerprinting of a broad range of phenolic compounds using high-throughput reversed-phase liquid chromatography coupled to robust and low-cost spectroscopic detection. This RP-UHPLC–DAD platform showed excellent performance in terms of linearity, sensitivity, matrix effect, accuracy, intra- and interday precision, specificity, and carryover for the identification and quantification of 69 plant phenolics in different food matrices (i.e., olive oil, red wine, strawberry) and urine samples. Of note, the use of an LC–DAD-based setup, often simpler, cheaper, and more commonly available than other instruments (e.g., mass spectrometry), facilitates its implementation in any analytical laboratory, either from the food industry or the research field. However, it is also remarkable that the chromatographic conditions optimized were compatible with MS detection, which would facilitate method migration to LC-MS systems for improved sensitivity detection and more confident identifications. The main limitation of the present study was the application of simple extraction protocols from the literature, which were devised to allow for large-scale screening of as many metabolites as possible, but in turn might have hindered the analysis of minor species. In this vein, the use of advanced sample treatment procedures (e.g., solid-phase extraction and preconcentration, enzymatic urinary hydrolysis) could maximize the potential of our RP-UHPLC–DAD platform in food and nutrimetabolomics research. Therefore, future studies are needed to get deeper insights into the characteristic phenolic profiles of the samples considered here as a case study and other food and biological matrices, as well as to investigate the factors that may influence polyphenol content. Furthermore, it should also be noted that method application to real food samples enabled the detection of numerous “unknown” metabolites that could tentatively be identified as phenolic compounds based on their characteristic UV/Vis spectra, which in turn might be semi-quantified using the calibration curves of analogous metabolites. In this respect, we would like to emphasize that the method presented here is not intended to be definitive but rather might undergo constant evolution by including new phytochemical standards with the aim of enlarging its metabolomics coverage.

## Supporting Information Available

Details about the preparation of standard solutions, method validation parameters, and concentrations of phenolic compounds detected in olive oil, red wine, strawberry, and urine samples (PDF)
