# Supplementary material for: High-Throughput Method for Wide-Coverage and Quantitative Phenolic Fingerprinting in Plant-Origin Foods and Urine Samples
Source: J Agric Food Chem. 2022 Jun 15;70(25):7796–804. doi: 10.1021/acs.jafc.2c01453 (PMC10550202; doi:10.1021/acs.jafc.2c01453)
Supplement: Supplementary file 1 — jf2c01453_si_001.pdf [file jf2c01453_si_001.pdf]

Supporting Information

High-Throughput Method for Wide-Coverage and Quantitative Phenolic Fingerprinting in Plant-Origin Foods and Urine Samples

Raúl González-Domínguez[1,2,*], Ana Sayago[1,2], María Santos-Martín[1,2], Ángeles Fernández-Recamales[1,2,*]

[1]Agrifood Laboratory, Faculty of Experimental Sciences, University of Huelva. 21007 Huelva, Spain. [2]International Campus of Excellence CeiA3, University of Huelva. 21007 Huelva, Spain.

*Corresponding authors: Dr. Raúl González-Domínguez, Tel. +34 959219975, email: raul.gonzalez@dqcm.uhu.es; Dr. Ángeles Fernández-Recamales, Tel. +34 959219958, email: recamale@dqcm.uhu.es

Table S1. Details about the preparation of standard solutions.

metabolite	Molecular formula	Stock solvent
Multi-metabolite solution A		
Benzoic acid	$C_7H_6O_2$	Methanol
4-Hydroxybenzoic acid	$C_7H_6O_3$	Methanol
3,4-Dihydroxybenzoic acid	$C_7H_6O_4$	Methanol
Vanillic acid	$C_8H_8O_4$	Methanol
Gallic acid	$C_7H_6O_5$	Methanol
4-O-Methylgallic acid	$C_8H_8O_5$	Methanol
Syringic acid	$C_9H_{10}O_5$	Methanol
4-Hydroxyphenylacetic acid	$C_8H_8O_3$	Methanol
3,4-Dihydroxyphenylacetic acid	$C_8H_8O_4$	Methanol
trans-Cinnamic acid	$C_9H_8O_2$	Methanol
o-Coumaric acid	$C_9H_8O_3$	Methanol
m-Coumaric acid	$C_9H_8O_3$	Methanol
p-Coumaric acid	$C_9H_8O_3$	Methanol
Caffeic acid	$C_9H_8O_4$	Methanol
Ferulic acid	$C_{10}H_{10}O_4$	Methanol
3-Caffeoylquinic acid	$C_{16}H_{18}O_9$	Methanol
Sinapic acid	$C_{11}H_{12}O_5$	Methanol
3-(4-Hydroxyphenyl)propionic acid	$C_9H_{10}O_3$	Methanol
4-Hydroxybenzaldehyde	$C_7H_6O_2$	Methanol
3,4-Dihydroxybenzaldehyde	$C_7H_6O_3$	Methanol
Vanillin	$C_8H_8O_3$	Methanol
Syringaldehyde	$C_9H_{10}O_4$	Methanol
4-Ethylphenol	$C_8H_{10}O$	Methanol

metabolite	Molecular formula	Stock solvent
4-Vinylphenol	C_8H_8O	Methanol
Eugenol	$C_{10}H_{12}O_2$	Methanol
Methoxyeugenol	$C_{11}H_{14}O_3$	Methanol
Furfural	$C_5H_4O_2$	Methanol
5-(Hydroxymethyl)furfural	$C_6H_6O_3$	Methanol
2,5-dimethyl-4-hydroxy-furanone	$C_6H_8O_3$	Methanol
2,5-dimethyl-4-methoxy-furanone	$C_7H_{10}O_3$	Methanol
Tyrosol	$C_8H_{10}O_2$	Methanol
Hydroxytyrosol	$C_8H_{10}O_3$	Methanol
Oleuropein	$C_{25}H_{32}O_{13}$	Methanol
Ellagic acid	$C_{14}H_6O_8$	1 M Sodium hydroxide
Catechin	$C_{15}H_{14}O_6$	Methanol
Epicatechin	$C_{15}H_{14}O_6$	Methanol
Epigallocatechin gallate	$C_{22}H_{18}O_{11}$	Methanol
Epicatechin gallate	$C_{22}H_{18}O_{10}$	Methanol
Multi-metabolite solution B		
Methylgallate	$C_8H_8O_5$	Methanol
Ethylgallate	$C_9H_{10}O_5$	Methanol
4-Methylcatechol	$C_7H_8O_2$	Methanol
trans-Resveratrol	$C_{14}H_{12}O_3$	Methanol/DMSO 75:25 (v:v)
trans-Resveratrol 3-O-glucoside	$C_{20}H_{22}O_8$	Methanol/DMSO 75:25 (v:v)
Naringenin	$C_{15}H_{12}O_5$	Methanol/DMSO 75:25 (v:v)
Naringenin 7-O-neohesperidoside	$C_{27}H_{32}O_{14}$	Methanol/DMSO 75:25 (v:v)
Hesperetin	$C_{16}H_{14}O_6$	Methanol/DMSO 75:25 (v:v)
Hesperetin 7-O-rutinoside	$C_{28}H_{34}O_{15}$	Methanol/DMSO 75:25 (v:v)

metabolite	Molecular formula	Stock solvent
Quercetin	$C_{15}H_{10}O_7$	Methanol/DMSO 75:25 (v:v)
Quercetin 3-O-galactoside	$C_{21}H_{20}O_{12}$	Methanol/DMSO 75:25 (v:v)
Quercetin 3-O-glucoside	$C_{21}H_{20}O_{12}$	Methanol/DMSO 75:25 (v:v)
Quercetin 3-O-rutinoside	$C_{27}H_{30}O_{16}$	Methanol/DMSO 75:25 (v:v)
Quercetin 3-O-rhamnoside	$C_{21}H_{20}O_{11}$	Methanol/DMSO 75:25 (v:v)
Kaempferol	$C_{15}H_{10}O_6$	Methanol/DMSO 75:25 (v:v)
Kaempferol 3-glucoside	$C_{21}H_{20}O_{11}$	Methanol/DMSO 75:25 (v:v)
Isorhamnetin	$C_{16}H_{12}O_7$	Methanol/DMSO 75:25 (v:v)
Isorhamnetin 3-O-glucoside	$C_{22}H_{22}O_{12}$	Methanol/DMSO 75:25 (v:v)
Morin	$C_{15}H_{10}O_7$	Methanol/DMSO 75:25 (v:v)
Luteolin	$C_{15}H_{10}O_6$	Methanol/DMSO 75:25 (v:v)
Apigenin	$C_{15}H_{10}O_5$	Methanol/DMSO 75:25 (v:v)
Multi-metabolite solution C		
Cyanidin	$C_{15}H_{11}O_6$	Methanol
Cyanidin 3-O-glucoside	$C_{21}H_{21}O_{11}$	Methanol
Malvidin	$C_{17}H_{15}O_7$	Methanol
Malvidin 3-O-glucoside	$C_{23}H_{25}O_{12}$	Methanol
Pelargonidin	$C_{15}H_{11}O_5$	Methanol
Pelargonidin 3-O-glucoside	$C_{21}H_{21}O_{10}$	Methanol
Peonidin	$C_{16}H_{13}O_6$	Methanol
Peonidin 3-O-glucoside	$C_{22}H_{23}O_{11}$	Methanol
Delphinidin 3-O-glucoside	$C_{21}H_{21}O_{12}$	Methanol
Petunidin 3-O-glucoside	$C_{22}H_{23}O_{12}$	Methanol
Internal standards		
2,6-Dimethoxybenzoic acid	$C_9H_{10}O_4$	Methanol

metabolite	Molecular formula	Stock solvent
Bisphenol A	$C_{15}H_{16}O_2$	Methanol

Multi-metabolite solutions A and B are analyzed using the RP-UHPLC-DAD method that was optimized for non-anthocyanin compounds, multi-metabolite solution C is analyzed using the RP-UHPLC-DAD method that was optimized for anthocyanin compounds.

Table S2. Method validation parameters, retention times (RT), and maximum of absorbance for the 69 target phenolic compounds in the different matrices under study (S, solvent; OO, olive oil; RW, red wine; ST, strawberry). Limits of quantification are expressed as mg L^{-1} for solvent solutions and red wine samples, and as mg kg^{-1} for strawberry and olive oil samples.

**phenolic compound**	**matrix**	**λ (nm)**	**RT (min)**	**linearity (mg L^{-1})**	**matrix effect (%)**	**accuracy (%)**	**LOQ (mg kg/L^{-1})**	**intra-day precision (%, N = 5)**	**inter-day precision (%, N = 15)**
Phenolic acids – Benzoic acids									
Benzoic acid (BA)	S	280	9.83	0.1-100	-	105.9	0.18	0.1	7.6
	OO		9.86		86.4	89.8	0.20	2.2	6.1
	RW		9.80		82.9	114.2	0.21	2.2	4.1
	ST		9.82		78.9	85.6	0.21	1.9	9.0
4-Hydroxybenzoic acid (4-HBA)	S	260	3.67	0.05-100	-	92.7	0.052	2.4	6.6
	OO		3.68		96.8	104.2	0.053	1.6	5.5
	RW		3.67		94.8	107.2	0.054	3.5	3.4
	ST		3.64		99.4	102.0	0.052	1.1	7.5
	S	260	2.10	0.05-100	-	98.0	0.053	2.4	6.9

**phenolic compound**	**matrix**	**λ (nm)**	**RT (min)**	**linearity (mg L^{-1})**	**matrix effect (%)**	**accuracy (%)**	**LOQ (mg kg/L^{-1})**	**intra-day precision (%, N = 5)**	**inter-day precision (%, N = 15)**
3,4-Dihydroxybenzoic acid (3,4-DHBA)	OO		2.11		116.0	94.2	0.050	0.9	6.5
	RW		2.08		95.9	92.7	0.055	3.0	3.3
	ST		2.10		85.0	84.0	0.059	0.9	8.0
Vanillic acid (VA)	S	260	7.01	0.05-100	-	93.6	0.077	0.3	6.0
	OO		7.02		94.5	104.5	0.080	0.2	5.9
	RW		7.02		85.8	88.9	0.084	6.0	6.8
	ST		7.03		91.9	102.7	0.082	3.2	11.7
Gallic acid (GA)	S	280	1.18	0.05-100	-	98.5	0.072	0.8	5.7
	OO		1.19		77.6	69.4	0.091	2.6	5.8
	RW		1.16		75.0	63.4	0.096	1.6	1.6
	ST		1.18		63.9	64.4	0.11	0.3	7.6
Methylgallate (MeGA)	S	280	5.09	0.01-100	-	98.0	0.015	6.2	5.8
	OO		5.12		98.1	97.2	0.015	2.8	6.7

phenolic compound	matrix	λ (nm)	RT (min)	linearity (mg L^{-1})	matrix effect (%)	accuracy (%)	LOQ (mg kg/L^{-1})	intra-day precision (%, N = 5)	inter-day precision (%, N = 15)
	RW		5.11		91.6	115.5	0.016	6.7	5.6
	ST		5.12		105.6	106.7	0.014	6.2	10.7
Ethylgallate (EtGA)	S	280	10.67	0.01-100	-	99.2	0.022	0.3	6.0
	OO		10.67		102.5	115.0	0.021	0.2	3.2
	RW		10.69		113.5	96.1	0.020	2.3	2.8
	ST		10.67		101.0	107.8	0.021	1.9	5.6
4-O-Methylgallic acid (4-MeGA)	S	260	16.03	0.05-100	-	92.9	0.057	0.3	6.8
	OO		16.05		95.3	100.9	0.059	0.5	5.5
	RW		16.05		91.5	116.0	0.061	2.7	5.0
	ST		15.99		94.0	102.3	0.060	1.0	8.4
Syringic acid (SYR)	S	280	10.14	0.05-100	-	92.5	0.055	0.2	5.9
	OO		10.15		93.8	100.5	0.057	0.4	5.7
	RW		10.17		97.7	102.3	0.055	1.7	3.0

**phenolic compound**	**matrix**	**λ (nm)**	**RT (min)**	**linearity (mg L^{-1})**	**matrix effect (%)**	**accuracy (%)**	**LOQ (mg kg/L^{-1})**	**intra-day precision (%, N = 5)**	**inter-day precision (%, N = 15)**
	ST		10.16		96.5	113.0	0.056	2.3	6.5
Phenolic acids – Phenylacetic acids									
4-Hydroxyphenylacetic acid (4-HPAA)	S	280	4.82	0.01-100	-	106.4	0.025	3.2	8.5
	OO		4.80		74.2	68.3	0.031	5.9	8.2
	RW		4.80		94.3	87.9	0.026	7.0	8.3
	ST		4.81		79.9	80.9	0.031	4.5	8.6
3,4-Dihydroxyphenylacetic acid (3,4-DHPAA)	S	280	2.59	0.01-100	-	96.3	0.028	0.5	4.1
	OO		2.61		85.4	82.4	0.033	4.8	7.5
	RW		2.57		105.7	108.6	0.027	5.8	9.6
	ST		2.59		65.7	75.6	0.039	2.5	7.2
Phenolic acids – Cinnamic acids									
trans-Cinnamic acid (tCA)	S	280	18.54	0.05-100	-	93.3	0.051	0.4	6.0
	OO		18.57		99.2	103.5	0.051	1.1	5.9

**phenolic compound**	**matrix**	**λ (nm)**	**RT (min)**	**linearity (mg L^{-1})**	**matrix effect (%)**	**accuracy (%)**	**LOQ (mg kg/L^{-1})**	**intra-day precision (%, N = 5)**	**inter-day precision (%, N = 15)**
	RW		18.59		99.3	110.6	0.051	1.9	1.9
	ST		18.57		90.3	98.7	0.053	0.6	8.9
o-Coumaric acid (oCOU)	S	280	14.40	0.05-100	-	93.0	0.064	0.3	5.9
	OO		14.44		94.5	103.0	0.066	0.7	5.6
	RW		14.45		99.4	102.5	0.064	3.3	3.9
	ST		14.45		86.4	115.5	0.073	2.0	7.4
m-Coumaric acid (mCOU)	S	280	12.76	0.05-100	-	92.8	0.047	0.3	6.0
	OO		12.79		94.5	103.0	0.048	0.5	5.8
	RW		12.81		93.9	110.6	0.049	2.2	2.2
	ST		12.78		89.7	113.9	0.051	0.4	6.6
p-Coumaric acid (pCOU)	S	320	11.05	0.05-100	-	92.6	0.053	0.3	5.9
	OO		11.07		93.9	102.1	0.056	0.1	5.5
	RW		11.09		94.3	102.0	0.056	2.5	2.8

**phenolic compound**	**matrix**	**λ (nm)**	**RT (min)**	**linearity (mg L^{-1})**	**matrix effect (%)**	**accuracy (%)**	**LOQ (mg kg/L^{-1})**	**intra-day precision (%, N = 5)**	**inter-day precision (%, N = 15)**
	ST		11.06		89.7	99.9	0.058	0.2	7.5
Caffeic acid (CA)	S	320	7.90	0.01-100	-	93.0	0.038	1.0	6.0
	OO		7.92		97.4	106.4	0.039	2.5	5.8
	RW		7.94		95.6	92.0	0.039	2.7	2.9
	ST		7.93		96.3	111.7	0.039	2.0	7.1
Ferulic acid (FA)	S	320	13.98	0.05-100	-	93.0	0.054	0.3	6.4
	OO		13.97		95.0	101.8	0.057	0.8	5.5
	RW		13.99		95.8	102.7	0.056	1.0	2.4
	ST		13.96		90.8	101.5	0.058	0.4	8.1
3-Caffeoylquinic acid (3-CQA)	S	320	10.63	0.05-100	-	91.3	0.048	0.3	5.3
	OO		10.65		94.5	98.0	0.050	0.2	4.8
	RW		10.68		91.6	95.5	0.051	2.7	2.6
	ST		10.66		95.6	99.8	0.050	1.3	5.1

phenolic compound	matrix	λ (nm)	RT (min)	linearity (mg L^{-1})	matrix effect (%)	accuracy (%)	LOQ (mg kg/L^{-1})	intra-day precision (%, N = 5)	inter-day precision (%, N = 15)
Sinapic acid (SIN)	S	320	15.73	0.05-100	-	93.3	0.049	0.4	3.0
	OO		15.75		101.6	100.9	0.049	1.6	3.8
	RW		15.76		105.3	92.8	0.048	6.0	10.5
	ST		15.74		110.5	112.5	0.047	3.9	10.2
Phenolic acids – Phenylpropionic acids									
3-(4-Hydroxyphenyl)propionic acid (4-HPPA)	S	280	8.72	0.05-100	-	112.1	0.050	4.8	10.9
	OO		8.70		80.2	77.2	0.062	5.2	11.6
	RW		8.75		88.7	91.9	0.057	5.3	6.3
	ST		8.75		81.3	79.8	0.060	1.7	11.1
Benzaldehydes									
4-Hydroxybenzaldehyde (4-HBAld)	S	280	5.06	0.05-100	-	93.5	0.046	1.2	6.1
	OO		5.06		95.5	102.8	0.046	1.2	5.5
	RW		5.07		93.5	100.4	0.047	2.5	4.2

**phenolic compound**	**matrix**	**λ (nm)**	**RT (min)**	**linearity (mg L^{-1})**	**matrix effect (%)**	**accuracy (%)**	**LOQ (mg kg/L^{-1})**	**intra-day precision (%, N = 5)**	**inter-day precision (%, N = 15)**
	ST		5.06		94.4	108.3	0.048	1.5	7.3
3,4-Dihydroxybenzaldehyde (3,4-DHBAld)	S	280	3.15	0.05-100	-	93.2	0.062	2.9	6.6
	OO		3.16		107.6	104.6	0.059	4.5	5.7
	RW		3.13		101.0	111.6	0.061	3.2	4.2
	ST		3.15		63.1	73.2	0.085	0.7	6.1
Vanillin (VAN)	S	280	8.93	0.05-100	-	91.2	0.059	2.2	3.6
	OO		8.93		106.2	98.5	0.056	0.9	3.9
	RW		8.98		104.5	103.1	0.056	3.9	7.1
	ST		8.90		96.3	96.2	0.060	0.9	3.8
Syringaldehyde (SYRAld)	S	280	11.99	0.05-100	-	92.3	0.044	0.3	5.9
	OO		11.99		94.7	104.3	0.045	0.4	7.1
	RW		12.02		95.1	110.7	0.045	3.8	4.4
	ST		11.99		88.8	101.4	0.048	4.5	9.6

phenolic compound	matrix	λ (nm)	RT (min)	linearity (mg L^{-1})	matrix effect (%)	accuracy (%)	LOQ (mg kg/L^{-1})	intra-day precision (%, N = 5)	inter-day precision (%, N = 15)
Phenols									
4-Methylcatechol (4-MeCAT)	S	280	4.89	0.05-100	-	95.7	0.067	3.4	10.0
	OO		4.91		96.8	101.3	0.068	2.8	5.5
	RW		4.92		100.5	106.3	0.067	6.1	5.1
	ST		4.88		93.4	98.4	0.069	2.7	5.9
4-Ethylphenol (4-EtPh)	S	280	17.54	0.1-100	-	104.2	0.13	4.7	9.8
	OO		17.54		90.8	109.4	0.14	8.1	13.5
	RW		17.56		95.8	86.1	0.14	7.1	11.2
	ST		17.54		105.9	106.8	0.13	7.1	11.8
4-Vinylphenol (4-VPh)	S	260	14.83	0.1-100	-	92.4	0.18	0.5	5.1
	OO		14.86		88.2	92.8	0.20	3.4	6.0
	RW		14.86		102.6	87.0	0.18	3.4	7.8
	ST		14.83		81.0	97.2	0.21	0.9	5.4

**phenolic compound**	**matrix**	**λ (nm)**	**RT (min)**	**linearity (mg L^{-1})**	**matrix effect (%)**	**accuracy (%)**	**LOQ (mg kg/L^{-1})**	**intra-day precision (%, N = 5)**	**inter-day precision (%, N = 15)**
Eugenol (EU)	S	280	22.89	0.1-100	-	90.5	0.11	1.8	7.1
	OO		22.91		95.2	111.1	0.11	2.9	6.9
	RW		22.94		89.4	100.3	0.12	5.2	7.9
	ST		22.92		87.1	100.3	0.12	2.8	8.5
Methoxyeugenol (MeEU)	S	280	23.83	0.1-100	-	97.6	0.33	2.8	6.5
	OO		23.84		90.2	108.6	0.36	6.2	7.9
	RW		23.86		83.7	96.9	0.38	6.2	8.7
	ST		23.85		90.8	98.1	0.37	9.4	10.3
Furan derivatives									
Furfural (F)	S	280	1.79	0.05-100	-	92.1	0.059	0.1	12.0
	OO		1.75		106.4	97.2	0.056	1.9	11.1
	RW		1.78		116.5	111.0	0.053	1.4	12.9
	ST		1.75		103.5	103.3	0.057	1.0	8.6

**phenolic compound**	**matrix**	**λ (nm)**	**RT (min)**	**linearity (mg L^{-1})**	**matrix effect (%)**	**accuracy (%)**	**LOQ (mg kg/L^{-1})**	**intra-day precision (%, N = 5)**	**inter-day precision (%, N = 15)**
5-(Hydroxymethyl)furfural (5-HMF)	S	280	1.51	0.05-100	-	98.7	0.066	1.0	5.1
	OO		1.51		70.4	64.2	0.093	1.8	5.3
	RW		1.48		103.7	102.6	0.066	1.7	2.4
	ST		1.50		63.8	72.2	0.098	1.9	6.9
2,5-dimethyl-4-hydroxy-furanone (DMHF)	S	280	2.33	0.05-100	-	97.5	0.048	0.4	8.7
	OO		2.35		95.0	82.6	0.050	3.7	7.5
	RW		2.38		117.3	116.8	0.043	2.2	4.4
	ST		2.32		88.2	116.7	0.053	4.5	10.2
2,5-dimethyl-4-methoxy-furanone (DMMF)	S	280	6.68	0.01-100	-	96.0	0.030	0.2	1.9
	OO		6.69		96.0	95.6	0.030	0.5	2.1
	RW		6.70		90.4	91.0	0.033	2.6	5.2
	ST		6.71		88.3	92.9	0.033	1.5	4.1
Phenylethanoids									

**phenolic compound**	**matrix**	**λ (nm)**	**RT (min)**	**linearity (mg L^{-1})**	**matrix effect (%)**	**accuracy (%)**	**LOQ (mg kg/L^{-1})**	**intra-day precision (%, N = 5)**	**inter-day precision (%, N = 15)**
Tyrosol (TYR)	S	280	3.87	0.1-100	-	87.6	0.085	2.5	11.3
	OO		3.87		89.4	90.5	0.094	3.4	9.2
	RW		3.84		92.6	102.3	0.092	2.9	4.9
	ST		3.90		91.8	92.4	0.092	4.9	10.2
Hydroxytyrosol (HTYR)	S	280	2.14	0.05-100	-	89.3	0.063	0.2	10.8
	OO		2.14		102.5	90.3	0.061	3.7	6.9
	RW		2.09		102.1	109.8	0.061	3.4	4.7
	ST		2.13		97.5	89.7	0.063	5.9	10.5
Oleuropein (OLE)	S	280	21.46	0.1-100	-	91.4	0.13	0.1	6.5
	OO		21.45		110.2	109.5	0.13	4.6	8.0
	RW		21.43		90.9	110.1	0.14	9.6	10.3
	ST		21.48		95.2	115.2	0.13	7.5	9.8
Tannins									

**phenolic compound**	**matrix**	**λ (nm)**	**RT (min)**	**linearity (mg L^{-1})**	**matrix effect (%)**	**accuracy (%)**	**LOQ (mg kg/L^{-1})**	**intra-day precision (%, N = 5)**	**inter-day precision (%, N = 15)**
Ellagic acid (EA)	S	260	18.90	0.5-100	-	97.9	0.60	0.9	8.8
	OO		18.90		109.2	85.2	0.55	0.7	5.3
	RW		18.90		79.9	83.6	0.65	3.7	4.1
	ST		18.88		105.4	87.1	0.57	0.5	5.6
Stilbenes									
trans-Resveratrol (tRSV)	S	320	20.23	0.01-100	-	96.9	0.021	0.1	6.2
	OO		20.26		101.0	107.8	0.021	0.1	6.3
	RW		20.28		107.6	110.9	0.020	4.7	4.4
	ST		20.25		113.6	116.9	0.020	0.6	7.0
trans-Resveratrol 3-O-glucoside (tRSV-3GLC)	S	320	17.28	0.01-100	-	97.0	0.0069	0.3	6.6
	OO		17.28		83.5	109.3	0.0082	0.3	7.1
	RW		17.31		88.3	109.7	0.0075	3.9	3.6
	ST		17.29		94.2	105.6	0.0070	3.7	8.8

**phenolic compound**	**matrix**	**λ (nm)**	**RT (min)**	**linearity (mg L^{-1})**	**matrix effect (%)**	**accuracy (%)**	**LOQ (mg kg/L^{-1})**	**intra-day precision (%, N = 5)**	**inter-day precision (%, N = 15)**
Flavonoids – Flavan-3-ols									
Catechin (C)	S	280	8.40	0.1-100	-	107.5	0.11	0.7	11.6
	OO		8.45		77.0	72.5	0.14	3.0	10.4
	RW		8.44		96.4	99.8	0.11	2.7	3.3
	ST		8.44		71.0	65.6	0.14	0.3	10.7
Epicatechin (EC)	S	280	12.44	0.1-100	-	93.0	0.11	0.2	3.6
	OO		12.47		97.5	101.9	0.11	1.7	4.0
	RW		12.47		94.5	102.2	0.11	7.1	5.9
	ST		12.46		80.3	82.5	0.12	0.3	4.9
Epigallocatechin gallate (EGCG)	S	280	13.70	0.05-100	-	101.4	0.066	0.3	8.9
	OO		13.73		111.3	85.6	0.064	5.3	7.9
	RW		13.74		119.5	99.8	0.064	3.9	8.2
	ST		13.73		117.8	103.4	0.065	1.2	6.8

phenolic compound	matrix	λ (nm)	RT (min)	linearity (mg L^{-1})	matrix effect (%)	accuracy (%)	LOQ (mg kg/L^{-1})	intra-day precision (%, N = 5)	inter-day precision (%, N = 15)
Epicatechin gallate (ECG)	S	280	17.35	0.1-100	-	93.5	0.11	2.1	6.8
	OO		17.37		98.6	100.9	0.11	4.4	8.4
	RW		17.37		100.0	82.2	0.11	4.4	6.9
	ST		17.35		97.5	82.3	0.11	3.3	10.6
Flavonoids – Flavanones									
Naringenin (NAR)	S	280	24.89	0.01-100	-	98.3	0.039	1.2	6.3
	OO		24.92		109.4	109.4	0.035	1.3	6.7
	RW		24.87		111.1	115.9	0.035	7.8	6.9
	ST		24.93		116.8	118.4	0.034	0.3	6.5
Naringenin 7-O-neohesperidoside (NAR-7NEO)	S	280	19.95	0.05-100	-	99.3	0.050	1.0	6.7
	OO		19.97		104.7	108.1	0.048	1.0	5.3
	RW		19.98		104.9	105.7	0.049	3.9	8.8
	ST		19.99		108.6	108.0	0.048	1.1	11.3

phenolic compound	matrix	λ (nm)	RT (min)	linearity (mg L^{-1})	matrix effect (%)	accuracy (%)	LOQ (mg kg/L^{-1})	intra-day precision (%, N = 5)	inter-day precision (%, N = 15)
Hesperetin (HES)	S	280	27.21	0.05-100	-	96.4	0.065	0.3	8.0
	OO		27.23		85.2	111.4	0.072	0.9	7.8
	RW		27.26		98.1	117.4	0.066	8.8	7.4
	ST		27.24		96.4	118.9	0.070	0.1	7.4
Hesperetin 7-O-rutinoside (HES-7RUT)	S	280	20.45	0.05-100	-	98.3	0.056	2.3	6.0
	OO		20.46		102.1	108.9	0.055	3.9	7.1
	RW		20.48		98.8	103.9	0.055	5.0	9.0
	ST		20.48		103.9	103.6	0.055	3.6	7.9
Flavonoids – Flavonols									
Quercetin (Q)	S	360	23.98	0.05-100	-	103.5	0.062	0.2	1.1
	OO		24.02		119.1	115.2	0.059	0.6	12.8
	RW		24.03		98.7	107.9	0.062	8.0	7.5
	ST		24.03		114.7	100.8	0.060	0.3	6.0

phenolic compound	matrix	λ (nm)	RT (min)	linearity (mg L^{-1})	matrix effect (%)	accuracy (%)	LOQ (mg kg/L^{-1})	intra-day precision (%, N = 5)	inter-day precision (%, N = 15)
Quercetin 3-O-galactoside (Q-3GAL)	S	360	19.10	0.01-100	-	94.3	0.020	0.1	6.5
	OO		19.12		87.0	105.2	0.022	0.3	7.0
	RW		19.12		94.4	110.4	0.020	5.3	5.7
	ST		19.12		96.0	116.2	0.020	0.1	6.1
Quercetin 3-O-rutinoside (Q-3RUT) / Quercetin 3-O-glucoside (Q-3GLC)	S	360	19.35	0.05-100	-	95.9	0.056	0.2	6.3
	OO		19.36		84.0	107.9	0.066	1.4	6.1
	RW		19.37		84.1	106.7	0.066	4.4	10.5
	ST		19.37		92.2	110.1	0.062	0.2	6.1
Quercetin 3-O-rhamnoside (Q-3RHA) / Kaempferol 3-glucoside (K-3GLC)	S	360	20.61	0.05-100	-	94.8	0.044	0.1	6.4
	OO		20.63		100.6	108.0	0.044	1.5	6.5
	RW		20.64		108.8	113.7	0.042	4.7	4.7
	ST		20.63		110.3	115.0	0.042	0.3	6.2
Kaempferol (K)	S	360	28.33	0.05-100	-	103.2	0.081	0.1	4.9

**phenolic compound**	**matrix**	**λ (nm)**	**RT (min)**	**linearity (mg L^{-1})**	**matrix effect (%)**	**accuracy (%)**	**LOQ (mg kg/L^{-1})**	**intra-day precision (%, N = 5)**	**inter-day precision (%, N = 15)**
	OO		28.35		118.7	126.7	0.080	0.2	5.1
	RW		28.38		107.5	103.5	0.081	8.9	8.4
	ST		28.36		124.4	126.6	0.081	0.2	7.0
Isorhamnetin (iR)	S	360	29.89	0.1-100	-	87.3	0.12	0.2	5.4
	OO		29.90		103.8	104.4	0.12	1.6	4.1
	RW		29.94		90.5	87.1	0.13	8.2	12.7
	ST		29.90		102.8	110.1	0.13	0.2	6.7
Isorhamnetin 3-O-glucoside (iR-3GLC)	S	360	21.13	0.05-100	-	86.1	0.042	0.1	6.5
	OO		21.14		101.1	97.3	0.042	1.2	7.0
	RW		21.16		105.1	100.3	0.041	6.9	6.4
	ST		21.16		111.8	111.5	0.042	0.3	6.6
Morin (MOR)	S	360	21.49	0.1-100	-	84.3	0.14	0.3	2.2
	OO		21.51		96.9	93.5	0.14	1.1	6.5

phenolic compound	matrix	λ (nm)	RT (min)	linearity (mg L^{-1})	matrix effect (%)	accuracy (%)	LOQ (mg kg/L^{-1})	intra-day precision (%, N = 5)	inter-day precision (%, N = 15)
	RW		21.53		106.6	114.5	0.13	6.4	5.7
	ST		21.52		119.8	125.5	0.12	1.4	7.0
Flavonoids – Flavones									
Luteolin (LUT)	S	360	25.14	0.1-100	-	102.5	0.10	0.1	6.0
	OO		25.16		117.1	124.8	0.085	0.2	6.5
	RW		25.12		100.5	120.8	0.10	8.4	9.3
	ST		25.18		101.1	109.5	0.098	0.5	6.3
Apigenin (API)	S	360	28.64	0.05-100	-	106.6	0.051	0.1	5.7
	OO		28.65		113.7	117.9	0.048	0.1	6.4
	RW		28.		110.8	104.8	0.049	9.4	9.9
	ST		28.66		114.3	118.7	0.049	0.2	6.1
Flavonoids – Anthocyanins									
Cyanidin (CYA)	S	520	9.64	1-50	-	79.0	3.51	2.6	7.5

**phenolic compound**	**matrix**	**λ (nm)**	**RT (min)**	**linearity (mg L^{-1})**	**matrix effect (%)**	**accuracy (%)**	**LOQ (mg kg/L^{-1})**	**intra-day precision (%, N = 5)**	**inter-day precision (%, N = 15)**
	RW		9.62		95.6	95.2	3.58	3.5	11.5
	ST		9.62		98.6	108.5	3.55	1.9	13.2
Cyanidin 3-O-glucoside (CYA-3GLC)	S	520	7.46	0.01-50	-	97.4	0.025	0.2	1.1
	RW		7.44		106.1	105.5	0.025	6.0	5.3
	ST		7.44		108.4	100.8	0.024	0.1	0.8
Malvidin (MAL)	S	520	12.52	1-50	-	77.1	1.22	0.5	13.7
	RW		12.52		84.8	101.7	1.33	6.4	9.9
	ST		12.52		120.3	116.0	1.21	2.2	10.1
Malvidin 3-O-glucoside (MAL-3GLC)	S	520	9.81	0.01-50	-	95.0	0.036	0.4	0.8
	RW		9.79		68.9	84.2	0.044	2.7	2.9
	ST		9.8		118.3	103.7	0.031	0.3	0.6
Pelargonidin (PEL)	S	520	10.83	1-50	-	81.5	1.74	0.6	5.3
	RW		10.86		97.2	88.0	1.78	2.2	8.5

**phenolic compound**	**matrix**	**λ (nm)**	**RT (min)**	**linearity (mg L^{-1})**	**matrix effect (%)**	**accuracy (%)**	**LOQ (mg kg/L^{-1})**	**intra-day precision (%, N = 5)**	**inter-day precision (%, N = 15)**
	ST		10.85		60.5	70.0	1.91	1.3	11.7
Pelargonidin 3-O-glucoside (PEL-3GLC)	S	520	8.18	0.05-50	-	101.9	0.047	0.2	1.2
	RW		8.18		111.2	111.3	0.048	6.1	5.0
	ST		8.16		100.8	102.3	0.045	0.1	0.6
Peonidin (PEO)	S	520	11.85	1-50	-	79.0	2.73	4.9	10.8
	RW		11.84		115.8	111.7	2.46	1.1	11.5
	ST		11.85		80.0	78.2	2.85	6.6	13.3
Peonidin 3-O-glucoside (PEO-3GLC)	S	520	9.08	0.01-50	-	97.9	0.030	0.4	1.2
	RW		9.07		109.6	104.4	0.027	5.5	4.8
	ST		9.08		117.0	106.1	0.026	0.2	0.8
Delphinidin 3-O-glucoside (DEL-3GLC)	S	520	6.68	0.01-50	-	96.3	0.028	0.2	1.1
	RW		6.66		102.3	98.2	0.030	4.5	4.2
	ST		6.66		110.7	99.6	0.025	0.5	1.0

phenolic compound	matrix	λ (nm)	RT (min)	linearity (mg L^{-1})	matrix effect (%)	accuracy (%)	LOQ (mg kg/L^{-1})	intra-day precision (%, N = 5)	inter-day precision (%, N = 15)
Petunidin 3-O-glucoside (PET-3GLC)	S	520	8.39	0.05-50	-	95.6	0.062	0.4	1.6
	RW		8.38		96.1	93.6	0.058	4.6	4.3
	ST		8.37		106.1	91.6	0.055	0.2	1.1
Internal standards									
2,6-Dimethoxybenzoic acid (2,6-DMBA)	S	280	9.44† / 5.10*					1.2† / 3.5*	6.9† / 4.1*
	OO		9.43†					0.5†	7.5†
	RW		9.42† / 5.10*					1.5† / 2.9*	5.7† / 2.8*
	ST		9.40† / 5.10*					0.3† / 3.6*	6.9† / 3.9*
Bisphenol A (BPA)	S	280	30.74† / 13.72*					3.5† / 2.1*	7.3† / 2.1*

phenolic compound	matrix	λ (nm)	RT (min)	linearity (mg L^{-1})	matrix effect (%)	accuracy (%)	LOQ (mg kg/L^{-1})	intra-day precision (%, N = 5)	inter-day precision (%, N = 15)
	OO		30.75†					2.4†	6.4†
	RW		30.81† / 13.73*					7.3† / 2.7*	7.4† / 2.2*
	ST		30.78† / 13.73*					1.5† / 2.7*	7.1† / 2.4*

For the internal standards, † refers to the retention times and validation parameters obtained for the RP-UHPLC-DAD method that was optimized for non-anthocyanin compounds, and * refers to the retention times and validation parameters obtained for the RP-UHPLC-DAD method that was optimized for anthocyanin compounds.

Table S3. Concentrations of phenolic compounds detected in olive oil, red wine, strawberry, and urine samples. The results are expressed as mean ± standard deviation of triplicate measurements (mg kg^{-1} for olive oil and strawberry, mg L^{-1} for red wine and urine). ND, non-detected.

	**olive oil**	**red wine**	**strawberry**	**urine**
phenolic acids - hydroxybenzoic acids				
4-Hydroxybenzoic acid (4-HBA)	0.64 ± 0.0058	ND	ND	3.62 ± 0.050
3,4-Dihydroxybenzoic acid (3,4-DHBA)	2.10 ± 0.019	2.03 ± 0.22	20.52 ± 0.95	4.32 ± 0.017
Vanillic acid (VA)	0.46 ± 0.035	13.65 ± 0.67	ND	ND
Gallic acid (GA)	ND	53.40 ± 0.99	20.01 ± 1.28	6.21 ± 0.41
Ethylgallate (EtGA)	ND	18.33 ± 0.70	ND	ND
4-O-Methylgallic acid (4-MeGA)	0.24 ± 0.011	ND	ND	ND
Syringic acid (SYR)	ND	7.68 ± 0.29	ND	2.28 ± 0.046
phenolic acids - hydroxyphenylacetic acids				
3,4-Dihydroxyphenylacetic acid (3,4-DHPAA)	ND	ND	31.77 ± 0.98	25.93 ± 1.26
phenolic acids - hydroxycinnamic acids				
trans-Cinnamic acid (tCA)	0.30 ± 0.0052	ND	10.33 ± 0.54	0.47 ± 0.0027
o-Coumaric acid (oCOU)	0.13 ± 0.0029	ND	ND	3.24 ± 0.089
m-Coumaric acid (mCOU)	0.41 ± 0.011	0.97 ± 0.072	ND	0.65 ± 0.039

	**olive oil**	**red wine**	**strawberry**	**urine**
p-Coumaric acid (pCOU)	0.24 ± 0.0019	6.54 ± 0.50	11.54 ± 0.20	0.40 ± 0.022
Caffeic acid (CA)	ND	9.53 ± 0.22	ND	0.28 ± 0.0018
Ferulic acid (FA)	0.14 ± 0.0029	ND	ND	ND
Sinapic acid (SIN)	ND	ND	ND	0.91 ± 0.13
hydroxybenzaldehydes				
3,4-Dihydroxybenzaldehyde (3,4-DHBAld)	ND	ND	12.40 ± 0.45	5.98 ± 0.54
Vanillin (VAN)	0.24 ± 0.0023	ND	ND	ND
phenols				
4-Ethylphenol (4-EtPh)	ND	16.38 ± 3.84	ND	ND
furan derivatives				
Furfural (F)	1.52 ± 0.062	1.15 ± 0.065	11.82 ± 0.25	ND
5-(Hydroxymethyl)furfural (5-HMF)	ND	0.44 ± 0.023	ND	ND
2,5-dimethyl-4-hydroxy-furanone (DMHF)	ND	ND	26.86 ± 6.17	ND
phenylethanoids				
Tyrosol (TYR)	12.25 ± 0.074	ND	ND	ND

	**olive oil**	**red wine**	**strawberry**	**urine**
Hydroxytyrosol (HTYR)	16.31 ± 0.42	ND	ND	ND
Oleuropein (OLE)	8.23 ± 0.65	ND	ND	ND
tannins				
Ellagic acid (EA)	ND	3308.51 ± 488.58	14194.67 ± 318.5	ND
stilbenes				
trans-Resveratrol (tRSV)	ND	1.84 ± 0.32	ND	ND
trans-Resveratrol 3-O-glucoside (tRSV-3GLC)	ND	2.30 ± 0.16	ND	ND
flavonoids - flavan-3-ols				
Catechin (C)	0.72 ± 0.042	21.23 ± 1.08	37.64 ± 1.13	ND
Epicatechin (EC)	ND	11.38 ± 0.30	45.43 ± 2.86	ND
Epicatechin gallate (ECG)	ND	2.77 ± 0.14	44.76 ± 1.03	ND
flavonoids - flavonols				
Quercetin (Q)	0.87 ± 0.027	9.38 ± 1.86	1.65 ± 0.071	ND
Quercetin 3-O-galactoside (Q-3GAL)	ND	ND	11.08 ± 0.36	ND
Quercetin 3-O-rutinoside (Q-3RUT) / Quercetin 3-O-glucoside (Q-3GLC)	ND	ND	17.25 ± 0.051	ND

	olive oil	red wine	strawberry	urine
Quercetin 3-O-rhamnoside (Q-3RHA) / Kaempferol 3-O-glucoside (K-3GLC)	ND	ND	8.91 ± 0.38	ND
Kaempferol (K)	ND	0.97 ± 0.19	1.02 ± 0.12	ND
falvonoids - flavones				
Luteolin (LUT)	6.95 ± 0.039	ND	ND	ND
Apigenin (API)	1.47 ± 0.028	ND	ND	ND
flavonoids - anthocyanins				
Cyanidin 3-O-glucoside (CYA-3GLC)	ND	1.52 ± 0.026	ND	ND
Malvidin 3-O-glucoside (MAL-3GLC)	ND	44.47 ± 0.033	ND	ND
Pelargonidin (PEL)	ND	ND	84.73 ± 5.71	ND
Pelargonidin 3-O-glucoside (PEL-3GLC)	ND	ND	62.48 ± 5.09	ND
Peonidin 3-O-glucoside (PEO-3GLC)	ND	4.40 ± 0.053	7.08 ± 0.67	ND
Delphinidin 3-O-glucoside (DEL-3GLC)	ND	11.07 ± 0.058	ND	ND
Petunidin 3-O-glucoside (PET-3GLC)	ND	13.17 ± 0.096	ND	ND
